# Barriers to learning and using point-of-care ultrasound: a survey of practicing internists in six North American institutions

**DOI:** 10.1186/s13089-020-00167-6

**Published:** 2020-04-19

**Authors:** Jonathan Wong, Steven Montague, Paul Wallace, Kay Negishi, Andrew Liteplo, Jennifer Ringrose, Renee Dversdal, Brian Buchanan, Janeve Desy, Irene W. Y. Ma

**Affiliations:** 1 Department of Medicine, University of Alberta, Edmonton, AB Canada; 2 Department of Medicine, Queen’s University, Kingston, ON Canada; 3 Department of Medicine, University of Pennsylvania, Philadelphia, PA USA; 4 Department of Medicine, Massachusetts General Hospital, Boston, MA USA; 5 Department of Emergency Medicine, Massachusetts General Hospital, Boston, MA USA; 6 Department of Medicine, Division of General Internal Medicine, Cumming School of Medicine, University of Calgary, 3330 Hospital Dr NW, Calgary, AB T2N 4N1 Canada; 7 Department of Medicine, Oregon Health & Science University, Portland, OR USA; 8 Department of Critical Care, University of Alberta, Edmonton, AB Canada

**Keywords:** Ultrasonography, Internal medicine, Continuing medical education, Barriers

## Abstract

**Background:**

Point-of-care ultrasound (POCUS) is increasingly used in internal medicine, but a lack of trained faculty continues to limit the spread of POCUS education. Using a framework based on organizational change theories, this study sought to identify barriers and enablers for hospital-based practicing internists to learn and use POCUS in clinical practice.

**Methods:**

We invited practicing internists at six North American institutions to participate in an electronic survey on their opinions regarding 39 barriers and enablers.

**Results:**

Of the 342 participants invited, 170 participated (response rate 49.3%). The top barriers were lack of training (79%), lack of handheld ultrasound devices (78%), lack of direct supervision (65%), lack of time to perform POCUS during rounds (65%), and lack of quality assurance processes (53%). The majority of participants (55%) disagreed or strongly disagreed with the statement “*My institution provides funding for POCUS training.*” In general, participants’ attitudes towards POCUS were favourable, and future career opportunities and the potential for billing were not considered significant factors by our participants in the decision to learn or use POCUS.

**Conclusions:**

This survey confirms the perceived importance of POCUS to practicing internists. To assist in closing faculty development gap, interventions should address training, supervision, quality assurance processes, availability of handheld devices, as well as dedicated time to perform POCUS during clinical care.

## Background

The use of point-of-care ultrasound (POCUS) is increasing in internal medicine (IM), not only for the guidance of bedside procedures [1–3], but also for bedside assessments of medical patients to answer focused clinical questions [4, 5]. In 2018, the American College of Physicians (ACP) officially acknowledged the important role of POCUS to improve diagnostic timeliness and care of the medical patient [6]. Following this acknowledgement from the ACP, the Society of Hospital Medicine issued a similar position statement highlighting their support for the use of POCUS by hospitalists [5]. Nationally and internationally, POCUS is increasingly incorporated in IM residency training programs over the last decade [7–11]. Recognizing the importance of POCUS in medical education, the Alliance of Academic Internal Medicine supports the integration of POCUS across the training continuum [12]. Despite the growing appetite for POCUS training, progress in expanding POCUS in IM has been slow. A major obstacle to the expansion of POCUS training is a lack of skilled faculty, and previous studies have cited limited time, money and resources as the key barriers to faculty adoption of POCUS [7, 11, 13–18]. A more nuanced understanding of perceived faculty barriers is needed, however, to allow for more targeted interventions.

Faculty adoption of POCUS requires significant behavioural change. Encouraging behavioural change is complex; it involves not only the individual practitioner in question (his/her motivation, aptitude, and attitudes), but also requires support within the organizational environment, such as proper leadership, policies, economics, and social and structural infrastructures [19–24]. It follows that any intervention to achieve system-level behavioural change will require a comprehensive, evidence-based strategy to identify and resolve barriers to POCUS adoption.

In this multi-centre survey study, we sought to identify IM faculty barriers and enablers to learning and using POCUS in clinical practice. Using a holistic approach based on organization change theories [19–21], we sought to explore both individual, as well as organizational barriers and enablers. The information obtained from this study will provide a deeper understanding of the barriers that IM faculty encounter to using POCUS to care for hospitalized patients.

## Methods

### Participants

In this multi-centre survey study, IM physicians caring for hospitalized patients were invited to complete an online survey. Six centres were chosen based on convenience, where site investigators indicated a reasonable likelihood of achieving an approximate target response rate of 40% or greater. The six study sites involved in this study were three centres in Canada (the University of Calgary in Calgary, Alberta; the University of Alberta in Edmonton, Alberta; Queen’s University in Kingston, Ontario) and three centres in the United States (Oregon Health & Science University in Portland, Oregon; Massachusetts General Hospital in Boston, Massachusetts; and the University of Pennsylvania in Philadelphia, Pennsylvania). Only consenting physicians were included. Physicians in Canadian centres were all internists or general internists certified by the Royal College of Physicians and Surgeons of Canada, with or without additional subspecialty certifications, and attended on hospital-based general medical teaching wards. Physicians in the United States were all board certified internists (or board eligible if in their first year of practice) who worked as hospitalists in their institutions. We excluded IM physicians practicing exclusively in an ambulatory care setting. Each study site sought and obtained local research ethics board approval for this study. The central coordinating site’s ethics approval was obtained from the Conjoint Health Research Ethics Board from the University of Calgary (Protocol REB 18-1498).

### Survey development

After reviewing key principles of organizational change theories [19–21], an online draft survey was developed by co-investigator (JW) and principal investigator (IM). The survey covered items on (1) demographics; (2) baseline POCUS experience; (3) individual barriers including emotional, clinical, financial factors; (4) group or social barriers; and (5) systemic or environmental barriers. This initial draft survey contained 11 questions regarding baseline demographics and POCUS experience information and 40 items on barriers and enablers. For each barrier and enabler, participants were asked to rate their agreement with the item on a 5-point Likert scale (from strongly disagree to strongly agree). The draft survey was piloted by 10 physician volunteers with a range in experience and knowledge of POCUS. These individuals were not part of the final participant pool. We sought their input on survey flow, wording, acceptability, administrative ease, question quality, and missing items. The survey was then revised based on their feedback. The final survey contained questions on baseline demographic and POCUS experience, and 39 items on barriers and enablers.

Between January and April 2019, participants were invited via email by the site investigator to complete the anonymized online survey administered using SurveyMonkey Inc. (San Mateo, California, USA).

### Statistical analysis

Standard descriptive statistics (mean, standard deviation, median, interquartile range) were calculated using SAS version 9.4 (SAS Institute Inc., Cary, NC, USA).

## Results

Of the 342 participants invited to complete the survey, 184 (53.8%) responded. Of these, 14 were excluded (declined consent *n* = 3; consented but did not complete the survey *n* = 11), resulting in a final pool of 170 participants (response rate 49.7%). Baseline characteristics of the participants are presented in Table 1.

**Table 1:** Baseline characteristics of 170 participants who completed the survey

Baseline characteristics	*N* (%)
Sex	
Male	95 (56)
Female	74 (44)
Age	
< 35 years	51 (30)
35–44 years	59 (41)
45–54 years	37 (22)
55–64 years	9 (5)
65 years or older	4 (2)
Years in clinical practice	
1–4 years	71 (42)
5–10 years	45 (26)
11–20 years	28 (16)
21 years or more	26 (15)
Prior point-of-care (POCUS) ultrasound training^a^	
No prior training	32 (19)
Self-learned (textbooks, YouTube, websites)	64 (38)
Bedside teaching (supervised scanning)	54 (38)
Didactic lecture(s)	55 (32)
< 1 day workshop/course	48 (28)
1–3 day workshop/course	61 (36)
Dedicated POCUS elective during training	17 (10)
POCUS-specific certifications	8 (5)
POCUS fellowship	4 (2)

Not every participant answered every question

^a^Participants were able to choose more than one response

### Clinical and procedural use

Participants reported using POCUS infrequently during clinical assessments of their patients (used on only a median of 5% of their patients, interquartile range 0–11%, range 0–90%). The majority of participants performed paracentesis in their practice (*n* = 126, 74%), whilst only a minority performed thoracentesis and central venous catheterization (*n* = 77, 45% and *n* = 66, 39%, respectively). For those who performed procedures, ultrasound was used for guidance *usually* or *always* for the majority of the participants (60/66 = 90% for central venous catheterization, 101/126 = 80% for paracentesis, and 63/77 = 82% for thoracentesis). A minority *never* used ultrasound for guidance when performing central venous catheterization (3/66 = 5%), paracentesis (7/126 = 6%), and thoracentesis (6/77 = 8%).

### Barriers to using POCUS

**Fig. 1:**
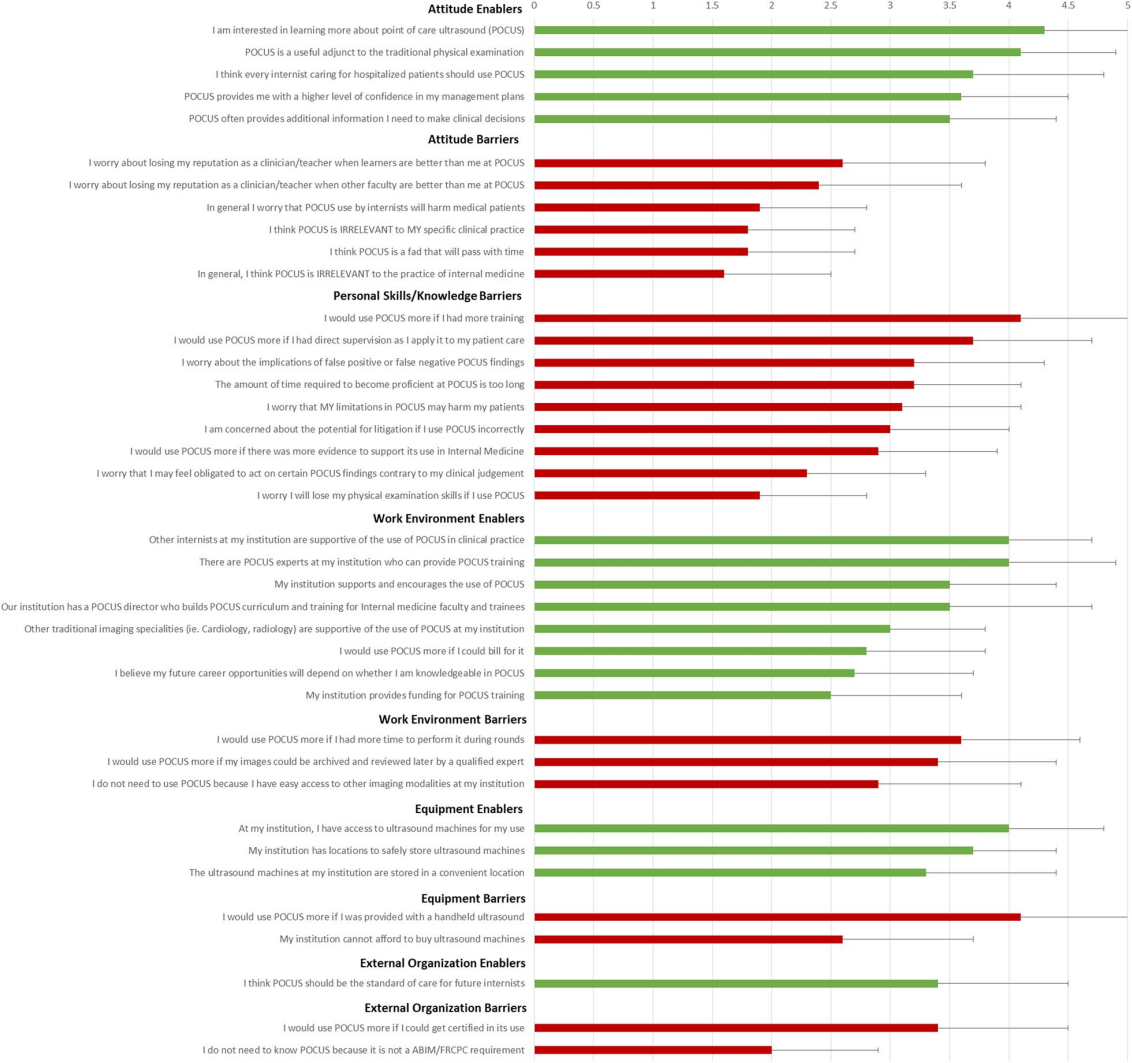
Barriers and enablers, presented in the descending order of agreement within each category. Barrier (red) and enablers (green) were rated on a 5-point Likert scale, where 1 = strongly disagree and 5 = strongly agree

Figure 1 outlines the results on the 39 barriers and enablers. The top 5 barriers were

1. Need for more training: 135/170, 79% agreed or strongly agreed with “*I would use POCUS more if I had more training*.”
2. Lack of handheld ultrasound device: 132/170, 78% agreed or strongly agreed with “*I would use POCUS more if I was provided with a handheld device*.”
3. Lack of direct supervision: 110/168, 65% agreed or strongly agreed with “*I would use POCUS more if I had direct supervision as I apply it to my patient care.*”
4. Lack of time to perform POCUS during rounds: 111/170, 65% agreed or strongly agreed with “*I would use POCUS more if I had more time to perform it during rounds.”*
5. Lack of quality assurance processes: 89/168, 53% agreed or strongly agreed with “*I would use POCUS more if my images could be archived and reviewed later by a qualified expert*.”

The 5 *least* important barriers appeared to be related to attitude regarding POCUS:

1. Irrelevance to internal medicine: 8/170, 5% agreed or strongly agreed with “*In general, I think POCUS is irrelevant to the practice of internal medicine*,” whilst 153/170, 90% disagreed or strongly disagreed with the statement.
2. POCUS is a fad: 7/170, 4% agreed or strongly agreed with “*I think POCUS is a fad that will pass with time*,” whilst 139/170, 82% disagreed or strongly disagreed.
3. Irrelevant to participant’s practice: 13/170, 8% agreed or strongly agreed with “*I think POCUS is irrelevant to my specific clinical practice*,” whilst 147/170, 86% disagreed or strongly disagreed.
4. Loss of physical examination skills: 10/170, 6% agreed or strongly agreed with “*I worry I will lose my physical examination skills if I use POCUS*,” whilst 135/170, 79% disagreed or strongly disagreed.
5. Patient harm: 10/170, 6% agreed or strongly agreed with “*In general, I worry that POCUS use by internists will harm medical patients*,” whilst 141/170, 83% disagreed or strongly disagreed.

### Enablers to using POCUS

Consistent with the lack of barriers regarding attitude against POCUS, data were triangulated with participants reporting an overall positive attitude towards the use of POCUS (e.g. high interest in learning more about POCUS, belief that POCUS is a useful adjunct to the traditional physical examination, Fig. 1, Table 2). The work environment (e.g. colleague attitudes, availability of POCUS experts) was overall seen as favourable to POCUS adoption. In general, participants reported a lack of funding for POCUS training, with 92/166 (55%) disagreed or strongly disagreed with the statement “*My institution provides funding for POCUS training.*” Future career opportunities and the potential for billing were not considered significant enablers by our participants (Fig. 1).

**Table 2:** Barriers and enablers, in the descending order of agreement

Attitude enablers	Mean (SD)^a^
I am interested in learning more about point-of-care ultrasound (POCUS)	4.3 (1.0)
POCUS is a useful adjunct to the traditional physical examination	4.1. (0.8)
I think every internist caring for hospitalized patients should use POCUS	3.7 (1.1)
POCUS provides me with a higher level of confidence in my management plans	3.6 (0.9)
POCUS often provides additional information I need to make clinical decisions	3.5 (0.9)

Attitude barriers	
I worry about losing my reputation as a clinician/teacher when learners are better than me at POCUS	2.6 (1.2)
I worry about losing my reputation as a clinician/teacher when other faculties are better than me at POCUS	2.4 (1.2)
In general I worry that POCUS use by internists will harm medical patients	1.9 (0.9)
I think POCUS is IRRELEVANT to MY specific clinical practice	1.8 (0.9)
I think POCUS is a fad that will pass with time	1.8 (0.9)
In general, I think POCUS is IRRELEVANT to the practice of internal medicine	1.6 (0.9)

Personal/general skills/knowledge barriers	
I would use POCUS more if I had more training	4.1 (0.9)
I would use POCUS more if I had direct supervision as I apply it to my patient care	3.7 (1.0)
I worry about the implications of false-positive or false-negative POCUS findings	3.2 (1.1)
The amount of time required to become proficient at POCUS is too long	3.2 (0.9)
I worry that MY limitations in POCUS may harm my patients	3.1 (1.0)
I am concerned about the potential for litigation if I use POCUS incorrectly	3.0 (1.0)
I would use POCUS more if there was more evidence to support its use in Internal Medicine	2.9 (1.0)
I worry that I may feel obligated to act on certain POCUS findings contrary to my clinical judgement	2.3 (1.0)
I worry I will lose my physical examination skills if I use POCUS	1.9 (0.9)

Work environment enablers	
Other internists at my institution are supportive of the use of POCUS in clinical practice	4.0 (0.7)
There are POCUS experts at my institution who can provide POCUS training	4.0 (0.9)
My institution supports and encourages the use of POCUS	3.5 (0.9)
Our institution has a POCUS director who builds POCUS curriculum and training for Internal medicine faculty and trainees	3.5 (1.2)
Other traditional imaging specialities (i.e. Cardiology, radiology) are supportive of the use of POCUS at my institution	3.0 (0.8)
I would use POCUS more if I could bill for it	2.8 (1.0)
I believe my future career opportunities will depend on whether I am knowledgeable in POCUS	2.7 (1.0)
My institution provides funding for POCUS training	2.5 (1.1)

Work environment barriers	
I would use POCUS more if I had more time to perform it during rounds	3.6 (1.0)
I would use POCUS more if my images could be archived and reviewed later by a qualified expert	3.4 (1.0)
I do not need to use POCUS because I have easy access to other imaging modalities (i.e. Chest X-rays, diagnostic ultrasound, CT, MRI, echocardiography, etc.) at my institution	2.9 (1.2)

Equipment-related enablers	
At my institution, I have access to ultrasound machines for my use	4.0 (0.8)
My institution has locations to safely store ultrasound machines	3.7 (0.7)
The ultrasound machines at my institution are stored in a convenient location	3.3 (1.1)

Equipment-related barriers	
I would use POCUS more if I was provided with a handheld ultrasound	4.1 (1.0)
My institution cannot afford to buy ultrasound machines	2.6 (1.1)

External organization-related enablers	
I think POCUS should be the standard of care for future internists	3.4 (1.1)

External organization-related barriers	
I would use POCUS more if I could get certified in its use	3.4 (1.1)
I do not need to know POCUS because it is not a ABIM/FRCPC requirement	2.0 (0.9)

^a^Rated on a 5-point Likert scale, where 1 = strongly disagree and 5 = strongly agree

## Discussion

In this multi-centre survey of six North American academic institutions’ practicing internists who look after hospitalized patients, several findings emerged. First, whilst POCUS is commonly used for procedural guidance, its frequency of use in the general clinical assessments was only done on a median of 5% of patients. Second, general attitudes towards POCUS were positive, including high interest in learning POCUS and an overall positive belief about the utility of POCUS. Third, practicing internists reported a number of barriers, including the lack of training, supervision, quality assurance processes (archiving and review of images), handheld devices, and time to perform POCUS during rounds. Whilst there was only moderate concern regarding the use of POCUS potentially resulting in patient harm, participants did not particularly feel that POCUS findings will compel them to act contrary to their clinical judgement, or that they may lose their physical examination skills if they used POCUS. Further, external factors such as career opportunities and billing opportunities were not considered significant enabling factors. Overall, our results suggest that the practicing internists at these six academic centres have limited personal, attitudinal, work environment, or externally related barriers to using POCUS but significant skill and knowledge-based barriers may be limiting POCUS use. Whilst access to machines did not seem to be a barrier at their institutions, participants did feel that provision of a handheld ultrasound would facilitate increased use.

Our results are consistent with prior survey studies on barriers in using POCUS in practicing physicians. For example, personal and general attitude to the use of POCUS has been favourable in prior studies [7, 11, 13, 14, 17, 25, 26]. However, unlike other studies where time, equipment, and funding were the primary barriers [7, 11, 16–18], our study participants were more concerned with their own lack of training, supervision, and the lack of quality assurance processes. This concern with lack of training and supervision has also been suggested in other studies [14, 15, 25, 26]. Last, in one study of neonatal and pediatric critical care specialists, over 40% of participants were concerned with both liability issues and resistance from imaging specialists [16]. These two issues were only of moderate importance to our survey participants.

Our study has several limitations. First is the issue of generalizability. The academic centres in our study all have a designated internal medicine POCUS champion, a marker of higher quality POCUS education [27], as well as availability of machines and supportive work environment and colleagues. As such, our results may not apply to practitioners in POCUS-naïve settings. Indeed, a prior study found that attitudes of critical care fellowship program directors differed between programs that had an ultrasound machine, compared with programs that did not [13]. Second, our overall response rate was only 49%. Whilst five of our six sites achieved our target of greater than 40% response rate, one site achieved only 33%. Nonetheless, our response rate is typical of studies of this kind; in a systematic review of 68 surveys, a mean response rate of 39.6% was reported [28]. Third, despite an attempt to more comprehensively explore barriers, a complete catalogue of all barriers is not possible. Fourth, whilst statistics convey central tendencies, for each barrier and enabler, the responses ranged from 1 to 5 (from *strongly disagree* to *strongly agree*), with the exception of 2 items: “*I do not need to know POCUS because it is not a ABIM/FRCPC requirement*” (range 1–4); “*Other internists at my institution are supportive of the use of POCUS in clinical practice”* (range 2–5). The implication of these observed ranges is that despite our reported results, in our study population are individuals whose attitude towards POCUS was very negative, who felt very strongly that POCUS is a fad that will pass with time, and who still would not use POCUS if they had more training and supervision. The usual interventions to effect behavioural change may be less effective on these individuals. Finally, our online survey was administered anonymously; despite the absence of identical responses in surveys, we cannot exclude the possibility that some individuals may have responded more than once.

## Conclusions

In conclusion, in our multi-centre survey of practicing hospital-based internists who look after hospitalized patients, lack of training, supervision, quality assurance processes (archiving and review of images), handheld devices, and time to perform POCUS during rounds were the key barriers to using POCUS. Personal attitudes about the utility of POCUS, current lack of requirement by external organizations for the practice of internal medicine, and inability to bill were not considered important barriers by our participants. Future studies should systematically address barriers to POCUS use.

## Data Availability

The datasets used and/or analyzed during the current study are available from the corresponding author on reasonable request.

## Abbreviations

ACP: American College of Physicians
IM: Internal medicine
POCUS: Point-of-care ultrasound
